# “Shielding” of Cytokine Induction by the Periodontal Microbiome in Patients with Periodontitis Associated with Type 2 Diabetes Mellitus

**DOI:** 10.32607/20758251-2019-11-4-79-87

**Published:** 2019

**Authors:** I. P. Balmasova, Y. A. Lomakin, E. A. Babaev, V. N. Tsarev, A. G. Gabibov, I. V. Smirnov, V. D. Knorre, L. A. Ovchinnikova, N. V. Gnuchev, E. N. Khurs, M. S. Deev, N. N. Kostin, S. D. Arutyunov

**Affiliations:** A.I. Evdokimov Moscow State University of Medicine and Dentistry of the Ministry of Healthcare of the Russian Federation, Moscow, 127473 Russia; Shemyakin-Ovchinnikov Institute of Bioorganic Chemistry RAS, Moscow, 117997 Russia; Engelhardt Institute of Molecular Biology, RAS, Moscow, 119991 Russia; Peoples’ Friendship University of Russia, Moscow, 117198 Russia

**Keywords:** chronic periodontitis, type 2 diabetes mellitus, interleukins, chemokines, bioplex, IL, periodontal pathogens, salivary cytokine profile

## Abstract

Periodontal diseases, especially those with polymicrobial etiology, are often associated with type 2 diabetes mellitus, proceeding more severely and affecting the course of diabetes mellitus. Recently, this feature has been associated with the ability of periodontopathogen microflora to cause not only a local infectious process in the oral cavity, but also to interact with the human immune system and induce various systemic effects. We investigated changes in the salivary cytokine profile of patients with chronic periodontitis, associated and not associated with type 2 diabetes mellitus. We observed a statistically significant decrease of MCP-1/CCL2, GM-CSF, IL-5, IL-6, and IFN-γ in the saliva of patients with chronic periodontitis associated with type 2 diabetes mellitus in comparison with patients with chronic periodontitis only. All of these cytokines are associated with macrophage activation. These data are an important contribution to the elucidation of the mechanism of periodontopathogens involvement in the manifestation of the systemic effects of type 2 diabetes.

## INTRODUCTION

Almost half of the world population is affected by oral diseases. Periodontal diseases initiated by bacterial species are especially significant, since they occur in people of all ages and ethnicities residing in any region [1, 2]. From 50 to 90% of all oral diseases are those that affect the periodontium [1, 3, 4]; approximately 7% of the world population has some severe form of chronic periodontitis [5].

A special feature of chronic periodontitis is that it has a polymicrobial etiology, as a certain stable bacterial community (periodontal pathogens) that has pronounced invasive properties is in a symbiotic relationship, and is capable of suppressing the immune response and promoting chronic inflammation [6, 7] (which becomes systemic with time [8, 9]) predominates in the periodontal biofilm.

Because of the systemic effects of periodontal pathogens that derive from their ability to persist in the human body within macrophages [10, 11], these pathogens are widely disseminated and are involved in the development of various systemic conditions [6, 12], such as infective endocarditis [13], atherosclerosis [14] and other cardiovascular diseases [15], bacterial pneumonia [16], obesity [17], diabetes mellitus [18], pregnancy outcomes [19], rheumatoid arthritis [20, 21], Alzheimer’s disease [22], inflammatory bowel disease [23], colon cancer [24], etc. Diabetes mellitus holds a prominent place among these disorders [18, 25].

Diabetes mellitus is one of the most common metabolic disorders [26]. As estimated by the International Diabetes Federation (IDF), the number of patients with diabetes mellitus will steadily increase to reach more than 500 million by 2030 [27]. Diabetes develops either because pancreatic β cells are unable to produce insulin or because peripheral tissues become insulin-resistant [28]. Therefore, two types of this disease have been singled out. Type 1 (insulin-resistant) diabetes is diagnosed in approximately 10% of all patients with diabetes mellitus and is associated with autoimmune destruction of pancreatic β cells, resulting in the body’s inability to produce insulin. Insulin-independent type 2 diabetes mellitus (90% of all diabetic patients) manifests itself in relative hyperinsulinemia caused by insulin resistance in cells [29]. Obesity and systemic inflammation are considered the shared risk factors for type 2 diabetes mellitus [30].

There are four major mechanisms for the pathogenesis of type 2 diabetes mellitus: hyperglycemia, insulin resistance, hyperlipidemia, and immune dysfunction [31]. The disorders caused by these mechanisms are tightly interrelated in the pathogenesis of obesity, inflammation, and diabetes mellitus. Chronic periodontitis fits well into this combination of pathological processes, since a high prevalence of periodontitis among all age groups is typical of patients with disorders of carbohydrate and lipid metabolism [29]. The key markers of type 2 diabetes mellitus are related very closely to the level of inflammatory cytokines and the severity of periodontal lesions in patients with chronic periodontitis [32, 33]. The severity of the inflammation in patients with different diseases can be assessed according to the changes in the cytokine profiles in the blood [34, 35], cerebrospinal fluid [36, 37], or saliva [38].

In patients with type 2 diabetes mellitus, local changes in the periodontium are characterized by increased production of reactive oxygen species and proinflammatory cytokines (IL-1, IL-6, and TNFα), as glycation products accumulate and become engaged in vigorous interaction with receptors. The increased levels of proinflammatory cytokines induce oxidative stress and subsequent periodontal tissue degradation [39].

There is ambiguity in the recent data regarding which of these diseases (type 2 diabetes mellitus or periodontitis) should be considered the underlying one and which one has a stronger impact on its comorbidity [40]. Thus, diabetic nephropathy and cardiovascular complications were reported to occur significantly more often in patients with type 2 diabetes mellitus associated with chronic periodontitis than in those without chronic periodontitis [41], while effective treatment of one of these comorbidities has a favorable impact on the course of the other one [42, 43]. This conception has also been proved in other studies showing that the systemic inflammatory status caused by pathogenic periodontal bacteria in patients with chronic periodontitis favors the development of type 2 diabetes [44]. It has been proved that proinflammatory cytokines play a considerable role in the appearance of insulin resistance [45] and are involved in the development of hyperlipidemia, one of the key pathogenetic signs of diabetes mellitus [46].

The objective of this study was to identify the characteristic features of the cytokine profile of the oral fluid of patients with comorbid chronic periodontitis and type 2 diabetes mellitus, using the clinical model of association between these pathological processes.

Both chronic periodontitis and type 2 diabetes mellitus are multifactorial disorders with rather diverse pathogenetic mechanisms, which make the development of sufficiently efficient experimental animal models a challenge.

Since periodontal disorders occur exceptionally rarely in animals, they are simulated by applying ligatures or using other traumatizing techniques. However, despite the large body of data that have been collected in animal experiments, in some cases it is extremely difficult to evaluate whether the results are applicable to humans, since today there is no simple, reproducible model that would actually mimic the pathogenesis of periodontal disorders in humans [47, 48].

The same can be said of the attempts to elaborate a robust experimental animal model of diabetes mellitus. For example, chemically and surgically induced models or genetically modified animals are used. However, it is believed that only separate aspects of the pathogenesis of diabetes mellitus can be studied using these models. Furthermore, most of the existing experimental models have failed to differentiate between types 1 and 2 diabetes mellitus [49, 50].

Because of the aforementioned problems, we used the clinical model which is based on the significant constraints imposed on the possible effect of individual symptoms of the diseases. Therefore, the study group involved patients older than 45 years who had moderate chronic periodontitis and type 2 diabetes mellitus complicated by stage 2 hypertension and had grade 1 or 2 obesity, since type 2 diabetes mellitus is almost always accompanied by complications.

## EXPERIMENTAL

**Patients with chronic periodontitis associated and not associated with type 2 diabetes mellitus and healthy subjects (controls)**

The clinical model used in this study involved three sex-matched groups of patients aged 45–60 years: (1) the study group consisting of 11 patients with chronic periodontitis associated with type 2 diabetes mellitus, (2) the comparison group consisting of 9 patients with chronic periodontitis, and (3) the control group consisting of 12 healthy donors. Patients with chronic periodontitis had no congenital maxillofacial anomalies; the papillary marginal and attached gingival (PMA) index was ≤ 60%, and the periodontal pocket depth was 3–5 mm (tooth mobility grade 1–2). The patients did not undergo any therapeutic interventions for a period of 6 months preceding the enrollment. The duration of type 2 diabetes mellitus was 3–10 years. All the patients were on oral antihyperglycemic therapy. The patients were monitored by controlling the blood level of glycated hemoglobin (HbA1c), the key criterion used to assess the quality of carbohydrate metabolism compensation in patients with diabetes mellitus [51]. The glycated hemoglobin (HbA1c) level during the observation period was 6.5–11.3%.

The study protocol was approved by the Ethics Committee of the A.I. Yevdokimov Moscow State University of Medicine and Dentistry (Ministry of Health of the Russian Federation). All the patients signed a written informed consent for participation in the research.

**Analysis of the cytokine profile in the oral fluid**

Whole unstimulated saliva samples (1 ml) were collected into sterile tubes. The saliva samples were stored at –80°C until further analysis. The unfrozen saliva samples were centrifuged at 16 100 *g *at 4°C for 10 min. The supernatant was diluted 2.5-fold with phosphate buffered saline (PBS) supplemented with 0.5% Tween 20.

The cytokine levels in saliva samples were determined by multiplex magnetic fluorescent immunoassay using a Human Cytokine 17-plex Assay kit on a Bio-Plex 200 system (Bio-Rad, USA) in accordance with the manufacturer’s recommendations. The cytokine levels were measured for a combination of 17 different cytokines, including (1) chemokines: interleukin 8 (IL-8), monocyte chemoattractant protein 1 (MCP-1), macrophage inflammatory protein 1β (MIP- 1β); (2) growth factors: granulocyte-colony stimulating factor (G-CSF), granulocyte-macrophage colony-stimulating factor (GM-CSF), interleukin 7 (IL-7); (3) proinflammatory cytokines: interleukin 1β (IL-1β), interleukin 6 (IL-6), tumor necrosis factor-α (TNFα), interleukin 17A (IL-17A); (4) cytokines related to humoral immunity: interleukins 4, 5, 13 (IL-4, IL-5, IL-13); (5) cytokines related to cell-mediated immunity: interleukins 2, 12 (IL-2, IL-12p70), interferon-γ (IFNγ); and (6) immunosuppressive cytokines: interleukin 10 (IL-10).

**Statistical analysis**

Data was analyzed using the SPSS (version 21) and Sigma-Plot 12.5 statistical software. The difference between patients and healthy subjects (controls) was compared by using the nonparametric Mann–Whitney U test. The differences were considered statistically significant at *p * < 0.05.

## RESULTS AND DISCUSSION

The levels of various cytokines in saliva samples were analyzed by comparing the 95% confidence intervals for the measured values and plotting the ROC curves, which show the ratio between the sensitivity and specificity for each test presented as the area under the curve (AUC) (see the diagrams).

**Fig. 1:**
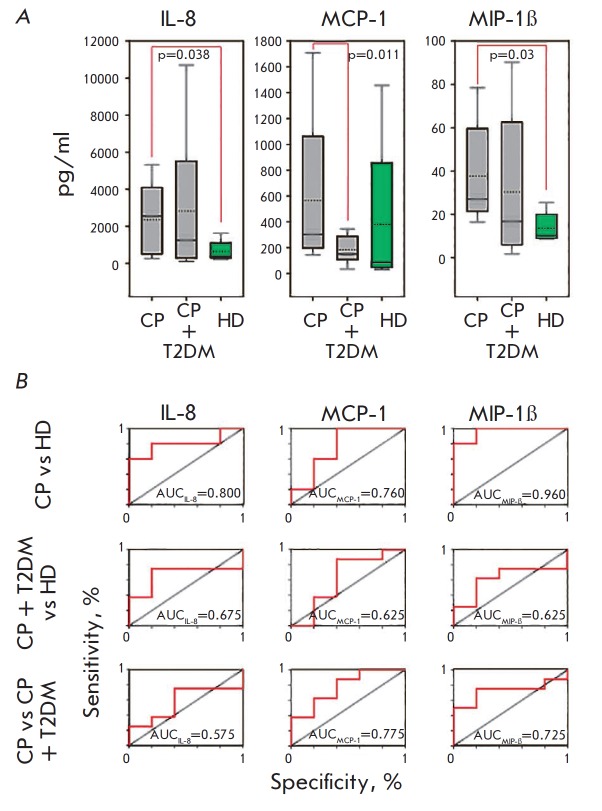
Levels of chemokines in the saliva (A) of patients with chronic periodontitis (CP), chronic periodontitis associated with type 2 diabetes (CP+T2DM), healthy donors (HD), and the corresponding ROC-curves (B). The interquartile range is shown by boxes. The median in each group is shown by the bold line. Bars represent the 95% confidence interval. Statistically significant differences with their respective *p *values are indicated; AUC – area under the ROC curve

*Figure 1* and *Table* show the results obtained in a comparative analysis of salivary chemokine levels and the ROC curves, indicating their diagnostic significance in all study groups. Comparison of 95% confidence intervals demonstrated that the salivary levels of such chemokines as IL-8 and the MIP-1β protein tended to increase in both groups of patients with chronic periodontitis; however, only in patients without type 2 diabetes mellitus is this increase statistically significant compared to that in the control group (the diagnostic significance of the test being rather high: AUC = 0.8–0.96). Meanwhile, no significant differences in elevation of the IL-8 level were revealed in the groups of patients with chronic periodontitis associated and not associated with type 2 diabetes mellitus (AUC = 0.574), while the MIP-1β levels could be assessed as being moderately high (AUC = 0.725). The salivary level of monocyte chemoattractant protein 1 (MCP1) was significantly lower in patients with chronic periodontitis associated with type 2 diabetes mellitus. In this case, the diagnostic significance of the test can be considered high (AUC = 0.775).

**Figure:**
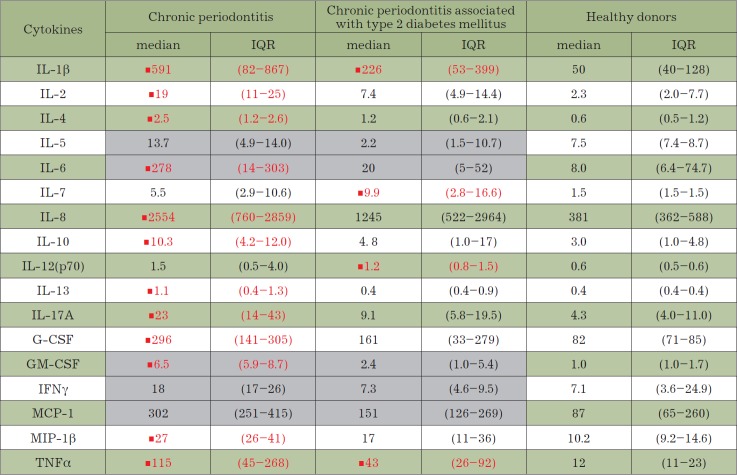
Table. The salivary cytokine profile of patients with chronic periodontitis associated and not associated with type 2 diabetes mellitus and healthy donors

Note. The results are presented as the median value and the interquartile range (IQR). Cytokine concentrations significantly differing from those in healthy donors (p < 0.05) are marked with the symbol “■” and shown in red color. Gray color denotes cytokine concentrations that differ significantly between the groups of patients with chronic periodontitis associated and not associated with type 2 diabetes mellitus (p < 0.05).

Anbalagan et al. [52] also reported that MCP-1 chemokine in the oral cavity has a special diagnostic significance in patients with type 2 diabetes mellitus. In particular, they emphasized that it is directly associated with the bacterial load in the oral cavity, since a reduction in the bacterial load due to therapeutic and

**Fig. 2:**
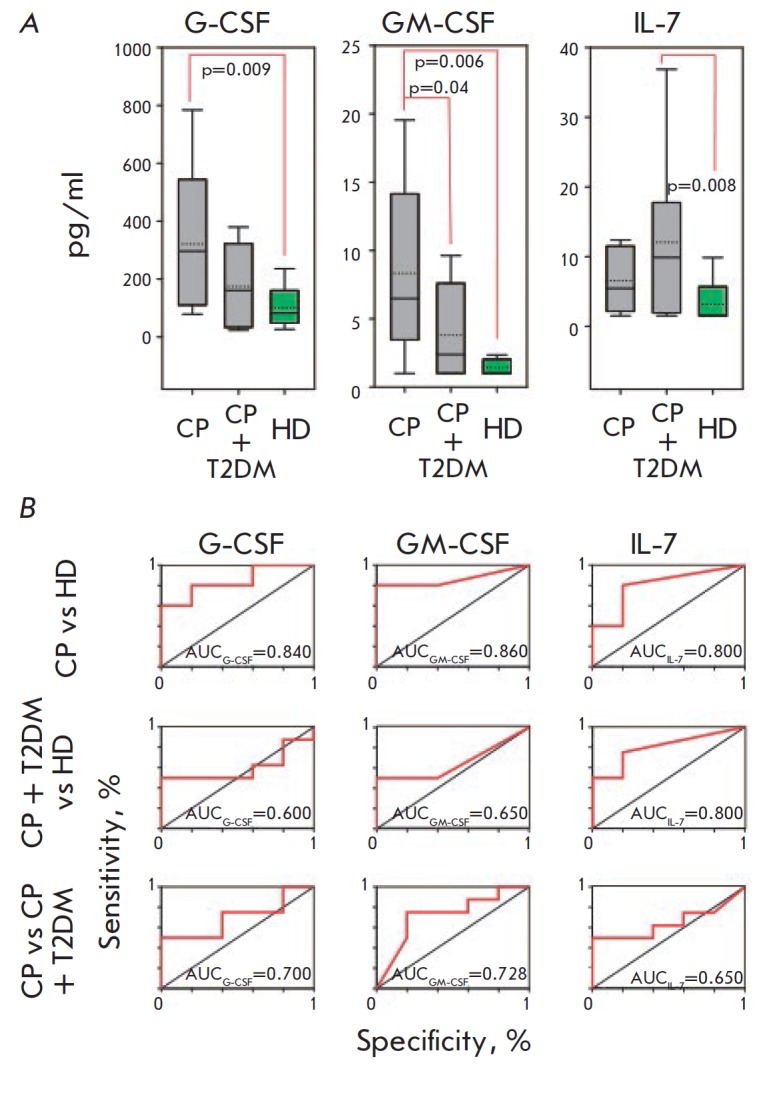
Levels of growth factors in the saliva (A) of patients with chronic periodontitis (CP), chronic periodontitis associated with type 2 diabetes (CP+T2DM) and healthy donors (HD), and the corresponding ROC-curves (B). The interquartile range is shown by boxes. The median in each group is shown by the bold line. Bars represent the 95% confidence interval. Statistically significant differences with their respective *p *values are indicated; AUC – area under the ROC curve

*Figure 2* and *Table* show the data obtained for a similar analysis of the salivary levels of several growth factors in patients and healthy subjects. The salivary G-CSF level in patients with chronic periodontitis tends to increase; however, this trend becomes a statistically significant deviation only in patients without type 2 diabetes mellitus. In all patients with chronic periodontitis, the salivary level of GM-CSF was higher than that in the control group. The IL-7 level was significantly high only in patients with chronic periodontitis associated with type 2 diabetes mellitus. Among these deviations, special attention should be paid to the differences between the GM-CSF levels in patients with chronic periodontitis associated and not associated with type 2 diabetes mellitus: they are higher in the latter group of patients.

Miranda et al. [55] demonstrated that in patients with chronic periodontitis associated with type 2 diabetes mellitus, the GM-CSF level (albeit in serum, not in saliva) is considered to be one of the pathogenetically important cytokines. Indirect evidence of the potential deficiency of this cytokine in patients with the comorbidities under study has been obtained, since exogenous administration of GM-CSF increases the survival rate of experimental animals [56].

**Fig. 3:**
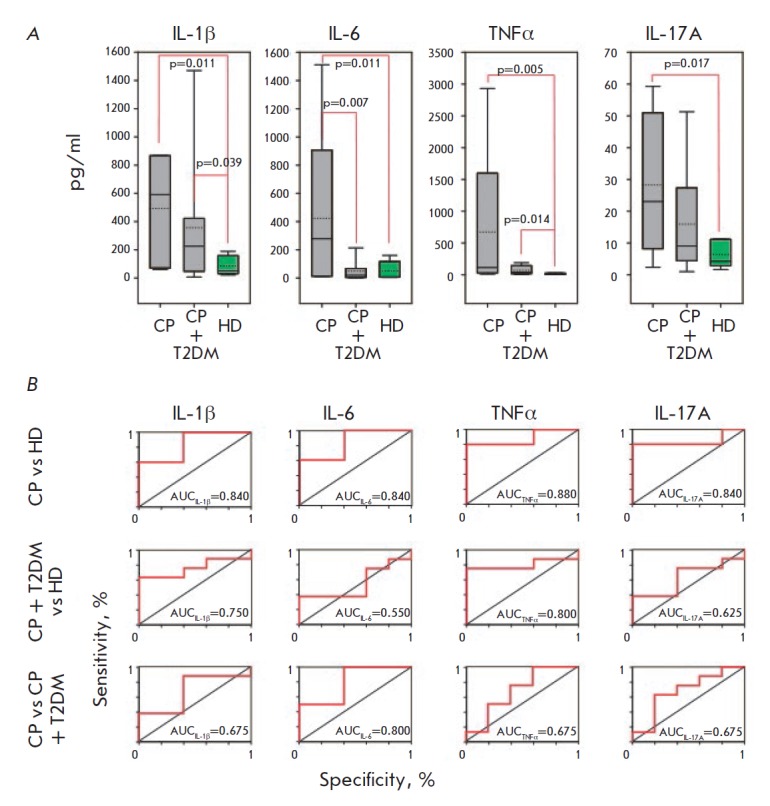
Levels of proinflammatory cytokines in the saliva (A) of patients with chronic periodontitis (CP), chronic periodontitis associated with type 2 diabetes (CP+T2DM) and healthy donors (HD), and the corresponding ROC-curves (B). The interquartile range is shown by boxes. The median in each group is shown by the bold line. Bars represent the 95% confidence interval. Statistically significant differences with their respective *p* values are indicated; AUC – area under the ROC curve

Next, the profile of four proinflammatory cytokines was analyzed: three of these cytokines are secreted mainly by innate immune cells (primarily by the macrophages IL-1β, IL-6, and TNFα), while IL-17A is a secretion product of Th17, one of the subtypes of T-helper cells (*Fig. 3*, *Table*).

*Figure 3* demonstrates that the salivary levels of IL-1β and TNFα were statistically significantly elevated in both groups of patients with chronic periodontitis regardless of whether or not they had comorbid type 2 diabetes. The IL-17A level was significantly increased in patients with chronic periodontitis, while only tending to increase in patients with both comorbidities.

The groups with chronic periodontitis associated and not associated with type 2 diabetes mellitus differed only in terms of the salivary level of IL-6, which was significantly elevated only in patients with chronic periodontitis with no comorbidity.

IL-6 is considered one of the key predictors of type 2 diabetes mellitus and its vascular complications. Assumptions have been made that the mechanism through which this cytokine is involved in the pathogenesis of atherosclerosis is systemic (via the activation of endothelial cells, due to the increasing role played by the thrombogenic function of platelets, via stimulation of proliferation of vascular smooth muscle cells, and due to the increased lipid accumulation in the macrophages) [57, 58]. We showed that the local salivary level of IL-6 is reduced in patients with chronic periodontitis associated with type 2 diabetes mellitus.

**Fig. 4:**
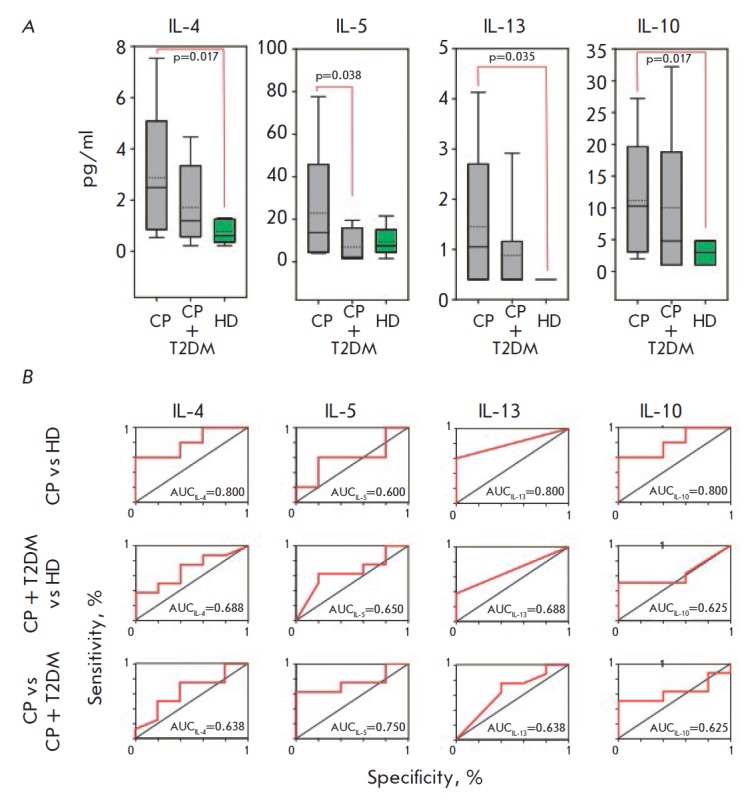
Levels of Th2 cytokines in the saliva (A) of patients with chronic periodontitis (CP), chronic periodontitis associated with type 2 diabetes (CP+T2DM) and healthy donors (HD), and the corresponding ROCcurves (B). The interquartile range is shown by boxes. The median in each group is shown by the bold line. Bars represent the 95% confidence interval. Statistically significant differences with their respective *p *values are indicated; AUC – area under the ROC curve

The levels of the cytokines that are secreted mainly by type 2 T-helper cells (Th2) and are to a certain extent associated with eliciting the humoral immune response were also determined (*Fig. 4*, *Table*).

Similar to other cytokines, the salivary levels of Th2- secreted IL-4, IL-13, and IL-10 in patients with chronic periodontitis associated with type 2 diabetes mellitus was higher than those in the control group, but lower than those in patients with chronic periodontitis without the comorbidity. However, these differences had a low diagnostic significance (AUC < 0.65). Only the levels of IL-5, which is secreted not only by Th2, but also by type 2 innate lymphocytes [59], differed in two groups of patients with chronic periodontitis (AUC = 0.75).

IL-5 is a growth factor that promotes eosin proliferation in adipose tissue, including in patients with type 2 diabetes mellitus [60]. Hypereosinophilia contributes to the transition of activated macrophages to the M2 phenotype, followed by suppression of inflammatory responses [60, 61]. As one of the components of this system (such as IL-5 in our study) is eliminated, the adipose tissue starts triggering insulin resistance (a typical feature of type 2 diabetes mellitus) and aggravating the inflammation [60, 62].

**Fig. 5:**
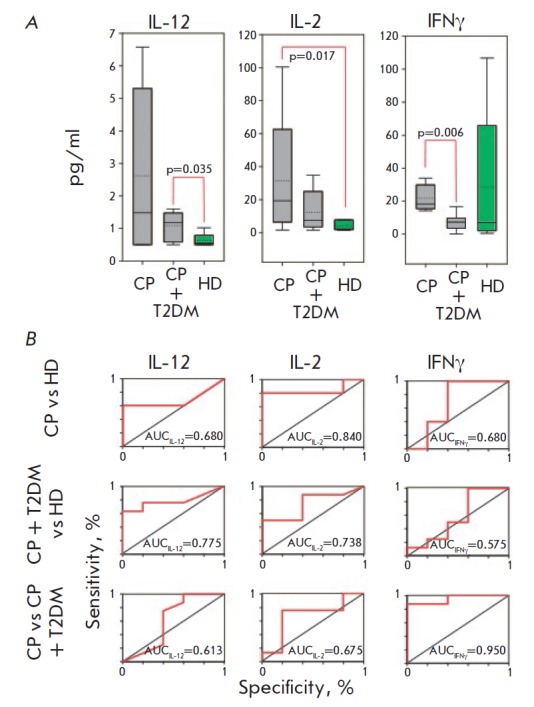
Levels of cell-mediated immunity cytokines in the saliva (A) of patients with chronic periodontitis (CP), chronic periodontitis associated with type 2 diabetes (CP+T2DM) and healthy donors (HD), and the corresponding ROC-curves (B). The interquartile range is shown by boxes. The median in each group is shown by the bold line. Bars represent the 95% confidence interval. Statistically significant differences with their respective *p* values are indicated; AUC – area under the ROC curve.

The measured levels of cytokines secreted by dendritic cells, macrophages, type 1 T-helper cells, and cytotoxic T cells are shown in *Figure 5* and *Table*. All of them (the active fraction of interleukin-12 (IL-12(p70)), IL-2, and interferon-γ (IFNγ)) are related to the eliciting of the cell-mediated immune response.

Significantly greater elevation of the salivary levels of IL-12 and IL-2 was observed in patients with chronic periodontitis (both associated and not associated with type 2 diabetes mellitus) compared to the control group; however, no differences between these groups were revealed. The salivary level of IFNγ suggests that IFNγ secretion is reduced in patients with chronic periodontitis; this reduction is greater when chronic periodontitis is associated with type 2 diabetes mellitus.

It has been reported that the serum IFNγ levels are reduced in patients with type 2 diabetes mellitus, especially during treatment [63]. Meanwhile, induction of the M1 macrophage phenotype is one of the functions of IFNγ [64].

*Table* summarizes the salivary cytokine levels in patients with chronic periodontitis (both associated and not associated with type 2 diabetes mellitus). It is obvious that the levels of 12 out of the 17 cytokines are significantly higher in patients with periodontitis compared to those in the control group. The levels differed for only five cytokines. Unambiguous differences between the groups of patients with chronic periodontitis associated or not associated with type 2 diabetes mellitus were established for the levels of only five cytokines.

An international research group with Russian participation has put forward an interesting hypothesis [65]. According to this hypothesis, hyperglycemia is the key factor in the pathogenesis of diabetes mellitus. Among all immune cells, an important role is played by macrophages whose activation is accompanied by a polarization of their functions, giving rise to two phenotypes: the classically activated M1 macrophages and the alternatively activated M2 macrophages. Due to the production of different cytokines, both of these phenotypes play a crucial role in the development of the inflammation and vascular complications associated with diabetes. Hyperglycemia per se (without allowance for additional effects) induces the mixed M1/M2 cytokine profile, which is responsible for the specific ratio between the inflammatory and vascular responses.

The observed features of the cytokine profile in patients with chronic periodontitis associated with moderate type 2 diabetes mellitus are apparently caused by an additional factor; chronic periodontitis etiologically related to the community of pathogenic periodontal bacteria that persist.

## CONCLUSIONS

The features of the local salivary cytokine profile typically observed in patients with chronic periodontitis associated with type 2 diabetes mellitus have been identified. These features were not observed in patients with chronic periodontitis not associated with diabetes and include statistically significant changes in the levels of MCP-1, GM-CSF, IL- 6, IL-5, and IFN-γ.

The key feature of the changes in the cytokine profile is the reduced secretion of the aforementioned cytokines, which is ground for assuming that the factor inducing cytokine secretion is "shielded" in patients with comorbid chronic periodontitis and type 2 diabetes mellitus. Pathogenic periodontal microflora etiologically related to chronic periodontitis can be such a factor.

Another important feature of the changes in the cytokine profile is the potential association between these deviations, the macrophage system, and the conditions required for macrophage activation. The combination of these features suggests that the selective effect of periodontal pathogens on the salivary cytokine profile is "shielded" as they switch to intracellular parasitism of macrophages, which subsequently elicits systemic effects.

## Abbreviations

IL: interleukin
TNFα: tumor necrosis factor-α
IFNγ: interferon gamma
MCP-1: monocyte chemoattractant protein-1
MIP-1β: macrophage inflammatory protein-1β
G-CSF: granulocyte-colony stimulating factor
GM-CSF: granulocyte-macrophage colony-stimulating factor
AUC: area under the ROC curve
ROC curve: receiver operating characteristic
